# An improved kilogram-scale preparation of atorvastatin calcium

**DOI:** 10.1186/s13065-015-0082-7

**Published:** 2015-02-13

**Authors:** Yuri V Novozhilov, Mikhail V Dorogov, Maria V Blumina, Alexey V Smirnov, Mikhail Krasavin

**Affiliations:** The Ushinsky Yaroslavl State Pedagogical University, 108 Respublikanskaya St., Yaroslavl, 150000 Russian Federation; Institute of Chemistry, St. Petersburg State University, 26 Universitetskyi Prospekt, Peterhof, 198504 Russian Federation

**Keywords:** Atorvastatin, Ketal deprotection, Ester hydrolysis, Hemi-calcium salt, Ethyl acetate solubility

## Abstract

**Background:**

If literature protocols are followed, conversion of an advanced ketal ester intermediate (available in kilogram quantities via a published Paal-Knorr synthesis) to cholesterol-lowering drug atorvastatin calcium is hampered by several process issues, particularly at the final stage where the hemi-calcium salt is obtained.

**Results:**

We developed a high-yielding synthesis of atorvastatin calcium salt on 7 kg scale that affords >99.5% product purities by introducing the following key improvements: i. isolating the pure product of the ketal deprotection step as crystalline solid, and ii. using a convenient ethyl acetate extraction procedure to isolate the pure atorvastatin calcium at the ester hydrolysis and counter-ion exchange step.

**Conclusion:**

The convenient and operationally simple conversion of an advanced intermediate of atorvastatin to the clinically used hemi-calcium salt form of the drug that is superior to the methods obtainable from the literature is now available to facilitate the production of atorvastatin calcium on industrial scale.

**Graphical abstract:**
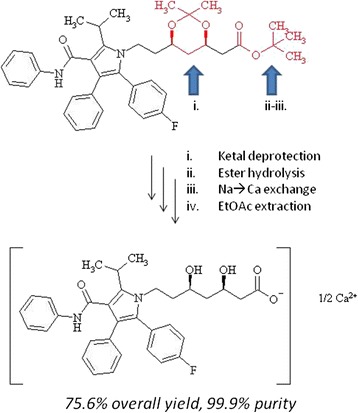
Stepwise ketal and tert-butyl ester group hydrolysis and a modified work-up protocol lead to a more convenient preparation of API-grade atorvastatin calcium.

**Electronic supplementary material:**

The online version of this article (doi:10.1186/s13065-015-0082-7) contains supplementary material, which is available to authorized users.

## Background

**Figure 1:**
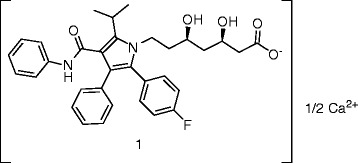
**Structure of Atorvastatin calcium.**

Atorvastatin calcium **1** is a known drug originally marketed by Pfizer under the trade name Lipitor (Figure 1) [1]. Atorvastatin inhibits HMG-CoA reductase and blocks the production of cholesterol in the body which helps control cholesterol levels in patients at risk of cardiovascular disease [2]. Following the expiration of the patent life of Lipitor in 2011 [3], the drug can be produced generically. This requires the development of efficient, non-proprietary production processes that would be available to a wide range of potential producers and enable the production of this important drug on an industrial, multi-kilogram scale. The importance of atorvastatin for public healthcare systems is accentuated, for example, by the fact that the drug was made part of the ‘Vital and Essential Drugs List” in the Russian Federation [4]. As part of a Government-funded initiative to promote local production of the active pharmaceutical ingredients for generic drugs, we engaged in the development of the industry-scale production of atorvastatin calcium. In this Communication, we disclose our findings on the improved synthesis of this drug that enable its multi-kilogram production.

## Results and discussion

**Scheme 1:**
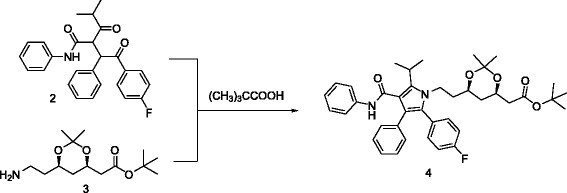
**Preparation of advanced atorvastatin precursor 4 via Paal-Knorr condensation.**

There are several known routes to atorvastatin which involve pyrrole ring construction either by [3 + 2] cycloaddition [5], Paal-Knorr condensation [5,6] or the Hantzsch pyrrole synthesis [7]. The most prominent synthesis, published over 20 years ago by Brower et al., involves condensation of the elaborated 1,4-diketone **2** (as a pyrrole precursor) with fully protected side chain amine **3** (Scheme 1) [8].

**Scheme 2:**
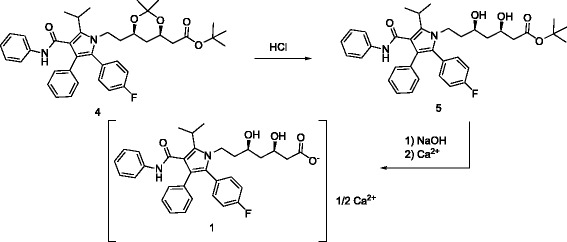
**General route for elaboration of 4 into atorvastatin hemi-calcium.**

This highly convergent route displays a number of attractive features: i. the high chemical yields (of the key pyrrole-forming step as well as en route to the synthons **2** and **3**), ii. only one step (toward **3**) requires substantial cooling, iii. the synthesis is scalable so as to yield multi-kilogram quantities of **4**, iv. there is no need to perform tedious and costly chromatographic purification in any of the steps toward **4**. All these features prompted us to select the route by Brower et al. as the basis for our kilogram-scale synthesis of atorvastatin calcium. However, elaboration of intermediate **4** amenable by this route into the final target material presented several issues when the published methods were applied. The conversion of the protected ketal – ester side chain into the requisite fully deprotected hemi-calcium salt form involves removal of the ketal protecting group under acid treatment, ester hydrolysis and counter-ion exchange (Scheme 2). Herein we describe a significantly improved method to convert advanced intermediate **4** into atorvastatin calcium on multi-kilogram scale.

The presence of the two acid-labile protecting groups in **4** (*gem*-dimethyl ketal for the diol and *tert*-butyl ester for the carboxylic acid) suggests that these two functionalities could be removed in a single step involving treatment with aqueous HCl, as it was previously reported [9-11]. However, in our hands, all attempts to bring about the simultaneous removal of the two protecting groups led to the formation of a number of unwanted and difficult-to-separate impurities. This was in accordance with the observations disclosed in an Indian patent [12] where HPLC analysis reportedly revealed the presence of at least five major impurities whose ratios depended on the reaction conditions. Teva Pharmaceuticals reported [13] that the bulk of impurities (e.g., atorvastatin lactone [7]) arose in the first step, i.e. the removal of the ketal protecting group (**4** → **5**). Therefore, it has been suggested [14,15] that the diol **5** is purified and obtained in crystalline form (as opposed to the oil form obtained by Teva [13]), prior to ester hydrolysis and hemi-calcium salt formation.

However, when we attempted to follow the published protocol in methanol [12,13], the quality of the product **5** obtained was below our expectations. Although its ^1^H NMR spectrum (see Additional file 1) could be regarded as adequate, the 2-3 impurities clearly detectable by TLC were worrisome. However, when the same de-ketalization of **4** with aqueous hydrochloric acid in isopropyl alcohol was brought about at 60°C, a 96% yield of >99% pure diol **5** was obtained on simple cooling and filtration. The use of isopropyl alcohol, in our view, provided a distinct advantage of our protocol. It afforded a fine, easy-to-filter solid, while when we tried the same reaction in aqueous acetonitrile [14,15], it resulted in a thick and much more difficult-to-filter precipitate of **5**.

Having achieved a clean and high-yielding conversion of **4** into **5**, we proceeded to study the conversion of the latter into atorvastatin calcium (**1**). Unfortunately, the available literature describing this step was somewhat cryptic about the details. It was clear, however, that the preparation of **1** could be achieved either by a strong alkaline solution of sodium hydroxide (in which case subsequent sodium-to-calcium counter-ion exchange will be required in a separate step) or directly, using calcium hydroxide. We found the latter option to be less convenient due to the low solubility of Ca(OH)_2_ in water or organic solvents, which inevitably resulted in the formation of a biphasic reaction system, longer reaction times and lower conversions. Therefore, we chose the two-step option for optimizing the process of converting **4** into **5**.

**Figure 2:**
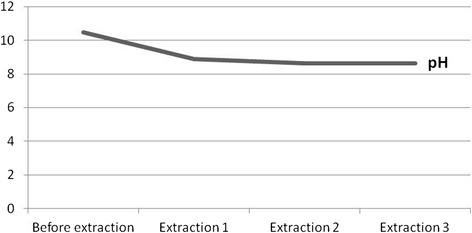
**pH of the atorvastatin sodium solution prior to and after repeated extractions with ethyl acetate.**

We found that the use of a large excess of sodium hydroxide in the hydrolysis was detrimental to the counter-ion swap step where it leads to the formation and precipitation of Ca(OH)_2_. A small excess of NaOH was still required to ensure a sufficient amount of this hygroscopic reagent was used. We found 1.10-1.15 equiv. of NaOH to be sufficient to achieve full 4 →5 conversions at 40°C in aqueous methanol. In order to neutralize the excess NaOH careful neutralization with acid was one option we considered. After some experimentation, we found that ethyl acetate added directly to the aqueous alkaline hydrolysis reaction mixture (in three repeats) not only efficiently quenched the excess sodium hydroxide (as judged by the marked drop in pH, Figure 2) it also allowed efficient extraction of unreacted **4** and other organic impurities possibly present in the crude reaction mixture, leaving behind an aqueous solution of atorvastatin sodium salt.

Moreover, we also found that ethyl acetate is useful for isolating the target compound (**1**) from the reaction mixture after hydrolysis and cation exchange. Interestingly, **1** has a high solubility in organic solvents while it is only sparingly soluble in water [13]. Therefore, after addition of calcium acetate to the solution of **1** obtained as described above, the target hemi-calcium salt is formed. The latter can be efficiently extracted from the aqueous solution, again, with ethyl acetate. The purity of **1** obtained after evaporation of the ethyl acetate extracts was over 95% and it was further improved (to 99.9%) by crystallization of the product from hot ethanol. The yield of **1** over two steps was in excess of 78%.

## Findings

In summary, we developed a highly efficient method for converting the advanced key intermediate **4** into atorvastatin calcium. The key feature of it is the use of ethyl acetate in the ester hydrolysis – calcium salt preparation step where the solvent plays three different roles: i. quenching the excess of NaOH, ii. removing the unreacted starting material (**4**) and other potential organic impurities from the reaction mixture, and iii. extracting the target compound **1** (as the hemi-calcium salt) from the aqueous solution. The solvents employed in these operations (methanol, ethanol and ethyl acetate) are much more environmentally benign compared to dichloromethane that had been earlier suggested [9] for precipitation of **1**.

## Methods

### *tert-*Butyl-(3*R*,5*R*)-7-[2-(4-fluorophenyl)-5-isopropyl-3-phenyl-4-(phenylcarbamoyl)pyrrol-1-yl]-3,5-dihydroxyheptanoate (5)

To a 100 L glass lined reactor isopropyl alcohol (70 L), water (17.5 L), **4** (7.0 kg, 11.38 mol) and 36% (d = 1.18 g/cm3) hydrochloric acid (0.8 L, 9.3 mol) were sequentially charged. The resulting suspension was heated, under vigorous stirring, to 60°C and kept at that temperature for 1 h. The solution was cooled to 5°C and stirred for 30 min. The precipitated solid was removed by centrifugation and washed with water (10 L). The resulting solid was dried at 50°C (0.3 bar) for 18 hours to provide 6.3 kg (96%) of **5**.

### Atorvastatin hemi-calcium salt (1)

Methanol (50 L), water (13 L) and sodium hydroxide (0.47 kg, 11.78 mol) in 100 L reaction vessel. To this solution **5** (6.3 kg, 10.25 mol) was added under vigorous stirring. The reaction temperature was raised and maintained at 40°C for 30 min. The progress of the reaction was monitored by TLC using 50% ethyl acetate in hexanes as eluent. On completion of the reaction, methanol was distilled from the reaction vessel at reduced pressure (about 40 L of the distillate was collected). To the residue, water (30 L) and ethyl acetate (15 L) were added and the resulting biphasic mixture was stirred for 30 min. The ethyl acetate layer was separated and the extraction procedure was repeated twice. To the resultant aqueous solution, ethyl acetate (30 L) was added and then calcium acetate monohydrate (1.08 kg, 6.15 mol) of was charged to the stirred biphasic mixture in one portion. After stirring for 40 min, the layers were separated and the bottom layer was discarded. The organic layer was washed twice with water-methanol mixture (95:5 v/v) and evaporated under reduced pressure. To the residue, 96% v/v ethyl alcohol (42 L) was added and the mixture was refluxed for 1 hour while precipitation was observed. The resulting suspension was cooled to 20°C over 3 hours and centrifuged. The product was separated, washed with 96% v/v ethanol (6 L), dried at 40°C (0.2 bar) for 12 hours to provide 4.66 kg (78.7% yield) of atorvastatin hemi-calcium salt of 99.9% purity.

## Description of additional material

‘Spectral (^1^H and ^13^C) NMR characterization data, copies of the respective spectra for compounds 4, 5 and 1, as well as a Quality Control (QC) report on a representative batch of atorvastatin calcium can be found online as Additional Material for the present article’.

## Additional file

Additional file 1:
**NMR spectra of compounds 4, 5, and 1 obtained in this study.**
