# Supplementary material for: An improved kilogram-scale preparation of atorvastatin calcium
Source: Chem Cent J. 2015 Feb 13;9:7. doi: 10.1186/s13065-015-0082-7 (PMC4333361; doi:10.1186/s13065-015-0082-7)
Supplement: Additional file 1: — NMR spectra of compounds 4, 5, and 1 obtained in this study. [file 13065_2015_82_MOESM1_ESM.pdf]

An improved kilogram-scale preparation of atorvastatin calcium

Yuri V. Novozhilov, Mikhail V. Dorogov, Maria V. Blumina, Alexey V. Smirnov and Mikhail Krasavin*

SUPPLEMENTARY DATA

- Spectral (^{1}H and ^{13}C) NMR characterization data
- Copies of ^{1}H and ^{13}C NMR spectra of compounds **4**, **5** and **1**
- QC report (HPLC) on a representative batch of atorvastatin calcium

Advanced intermediate 4: ^{1}H NMR (400 MHz, DMSO-d_6) δ ppm 9.77 (m, 1 H), 7.51 (d, *J*=7.89 Hz, 2 H), 7.23 (m, 6 H), 7.08 (m, 4 H), 7.00 (m, 2 H), 4.11 (br. s., 1 H), 3.93 (m, 1 H), 3.77 (m, 2 H), 3.30 (m, 2 H), 3.22 (quin, *J*=6.91 Hz, 1 H), 2.30 (m, 1 H), 2.17 (m, 1 H), 1.57 (m, 2 H), 1.37 (m, 15 H), 1.31 (s, 3 H), 1.17 (s, 3 H), 0.93 (q, *J*=12.06 Hz, 1 H); ^{13}C NMR (d_6-DMSO, 75 MHz) 169.94, 166.52, 163.75, 160.50, 139.90, 136.49, 135.31, 133.95, 129.63, 128.88, 128.08, 127.82, 125.86, 123.43, 121.09, 119.89, 118.03, 115.95, 115.67, 98.46, 80.19, 66.38, 66.16, 42.48, 38.24, 35.69, 30.25, 28.23, 26.10, 22.82, 20.09.

***tert*-Butyl-(3R,5R)-7-[2-(4-fluorophenyl)-5-isopropyl-3-phenyl-4-(phenylcarbamoyl)pyrrol-1-yl]-3,5-dihydroxyheptanoate (5).** ^{1}H NMR (400 MHz, DMSO-d_6) δ ppm 9.77 (s, 1 H), 7.51 (d, *J*=7.89 Hz, 2 H), 7.21 (m, 6 H), 7.07 (s, 4 H), 6.99 (d, *J*=7.23 Hz, 2 H), 4.66 (d, *J*=5.26 Hz, 1 H), 4.60 (d, *J*=4.82 Hz, 1 H), 3.94 (m, 1 H), 3.78 (m, 2 H), 3.51 (m, 1 H), 3.23 (quin, *J*=6.91 Hz, 1 H), 2.21 (m, 2 H), 1.35 (m, 19 H); ^{13}C NMR (d_6-DMSO, 75 MHz) 170.92, 166.58, 163.68, 160.44, 139.91, 136.46, 135.38, 133.87, 129.63, 128.88, 128.08, 127.79, 125.83, 123.42, 121.09, 119.88, 117.98, 115.96, 115.67, 79.92, 66.33, 65.90, 62.49, 44.09, 43.96, 28.28, 25.96, 22.75.

Atorvastatin hemi-calcium salt (1). ^{1}H NMR (400 MHz, DMSO-d_6) δ ppm 9.82 (s, 1 H), 7.51 (d, *J*=7.89 Hz, 2 H), 7.22 (m, 6 H), 7.07 (m, 4 H), 6.99 (m, 2 H), 3.95 (m, 1 H), 3.76 (m, 2 H), 3.53 (br. s., 1 H), 3.22 (quin, *J*=6.91 Hz, 1 H), 2.05 (dd, J_1=15,22 Hz, J_2=4,25 1 H), 1.91 (m, 1 H), 1.57 (m, 2 H), 1.39 (m, 7 H), 1.22 (m, 1 H); 178.56, 166.63, 163.67, 160.41, 139.92, 136.43, 135.38, 133.89, 133.78, 129.62, 128.88, 128.08, 127.77, 125.81, 123.43, 121.04, 119.88, 117.95, 115.97, 115.69, 66.76, 44.41, 44.15, 26.13, 22.76.

Compound **4**: ^{1}H NMR spectrum

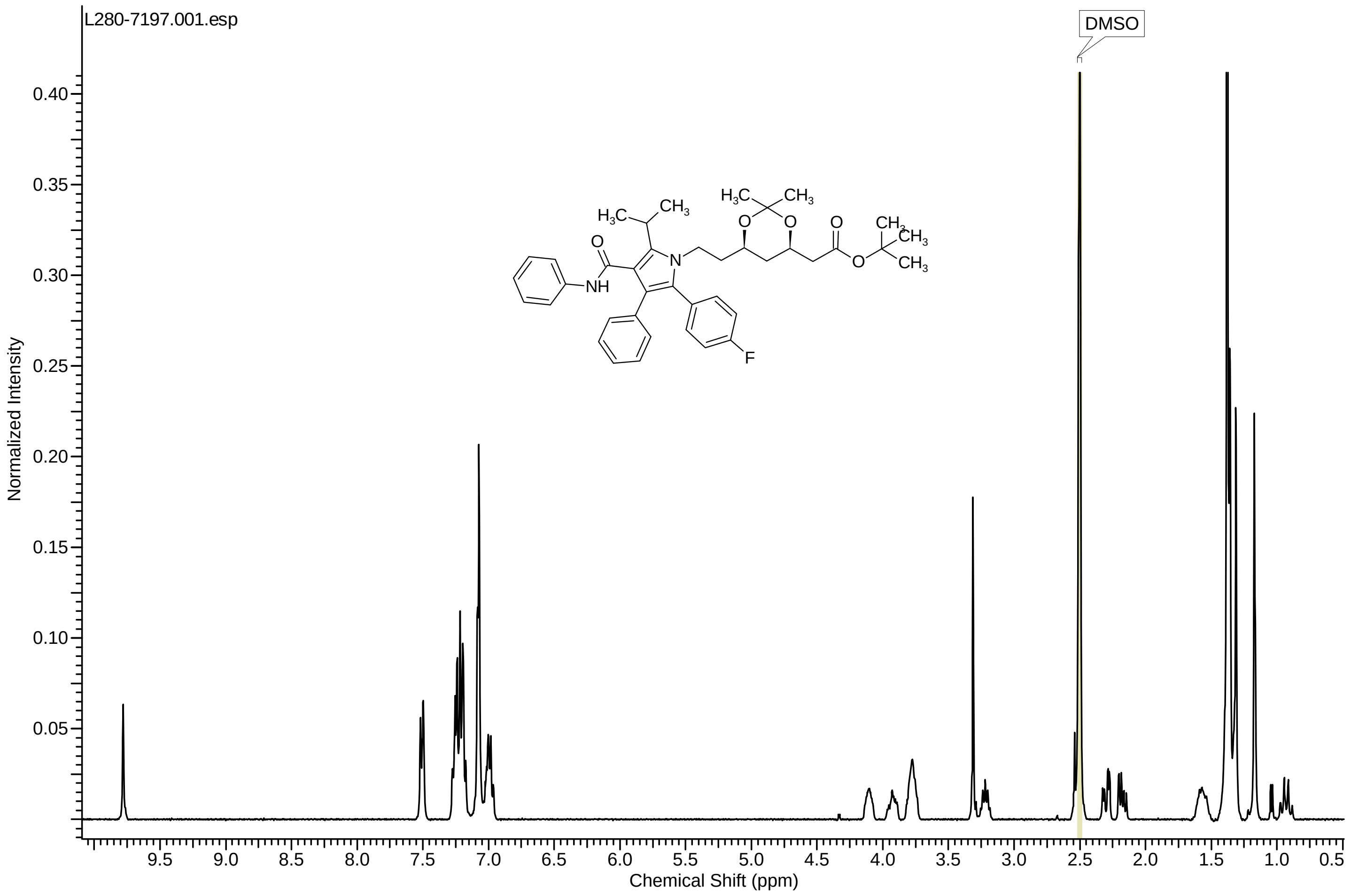

Compound **4**: ^{13}C NMR spectrum.

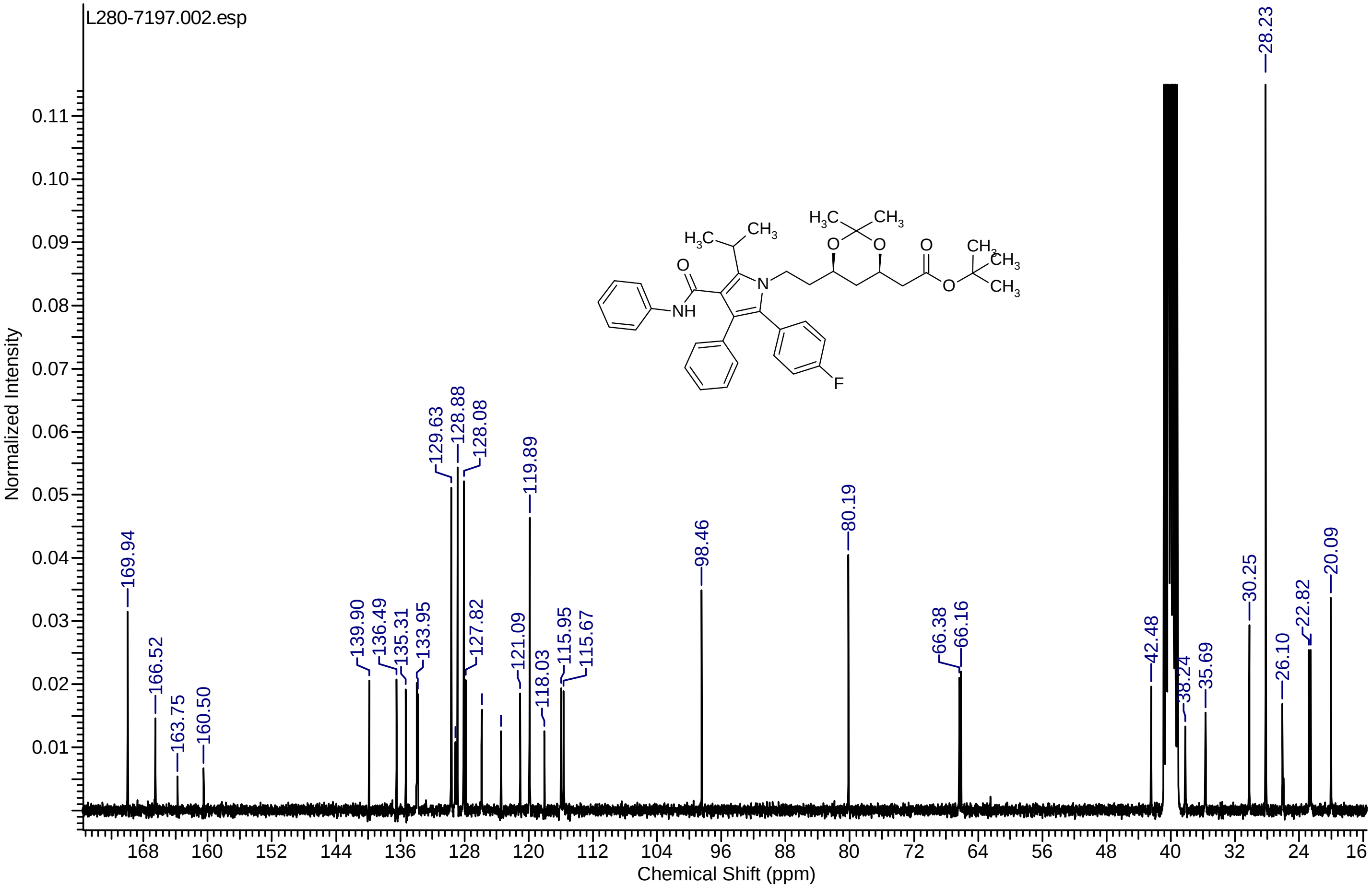

Compound **5**: ^{1}H NMR spectrum (ketal removal in isopropyl alcohol, as developed in this work)

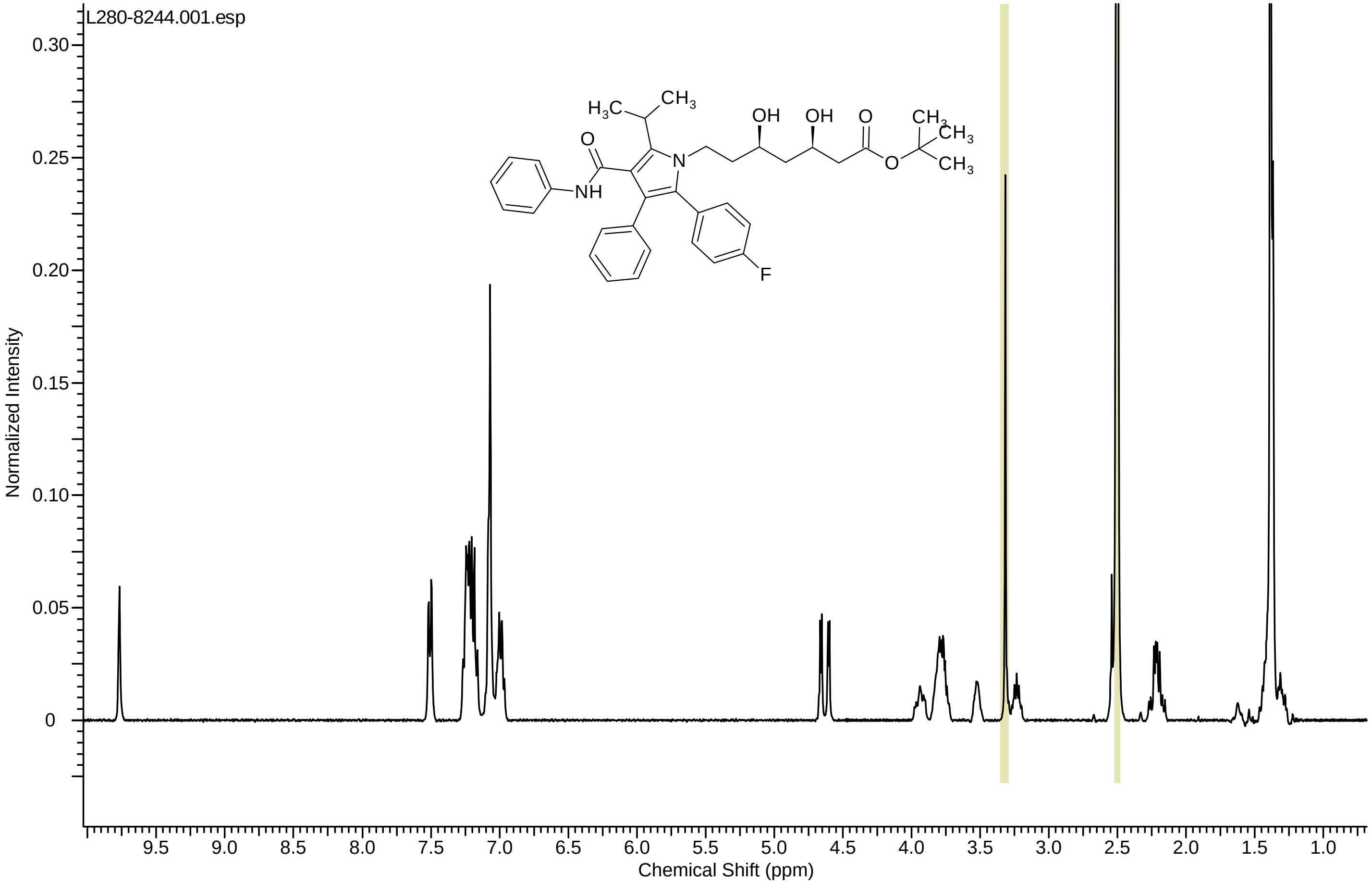

Compound **5**: ^{1}H NMR spectrum (ketal removal in methanol, attempted as described in refs 13-14)

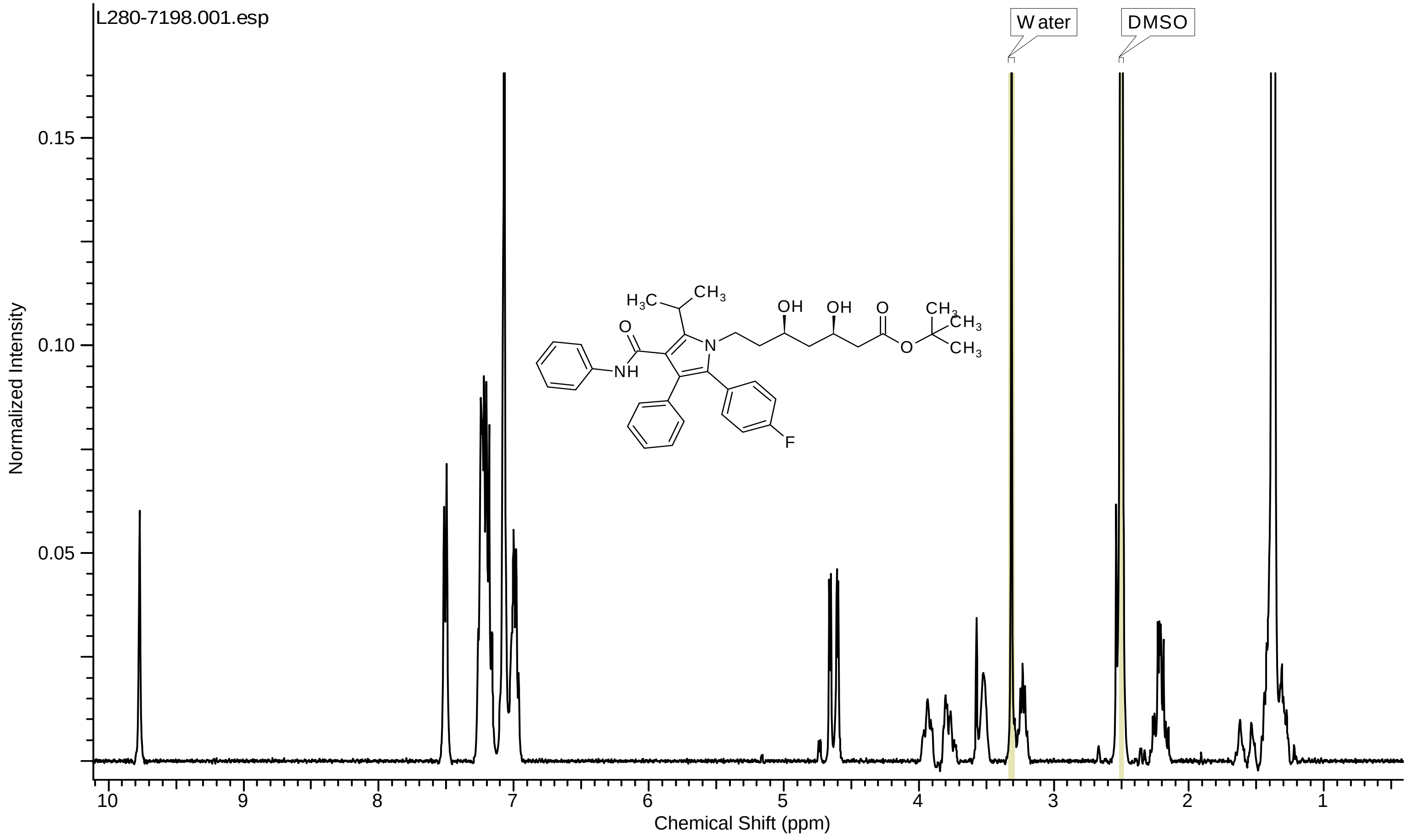

Compound **5**: ^{13}C NMR spectrum.

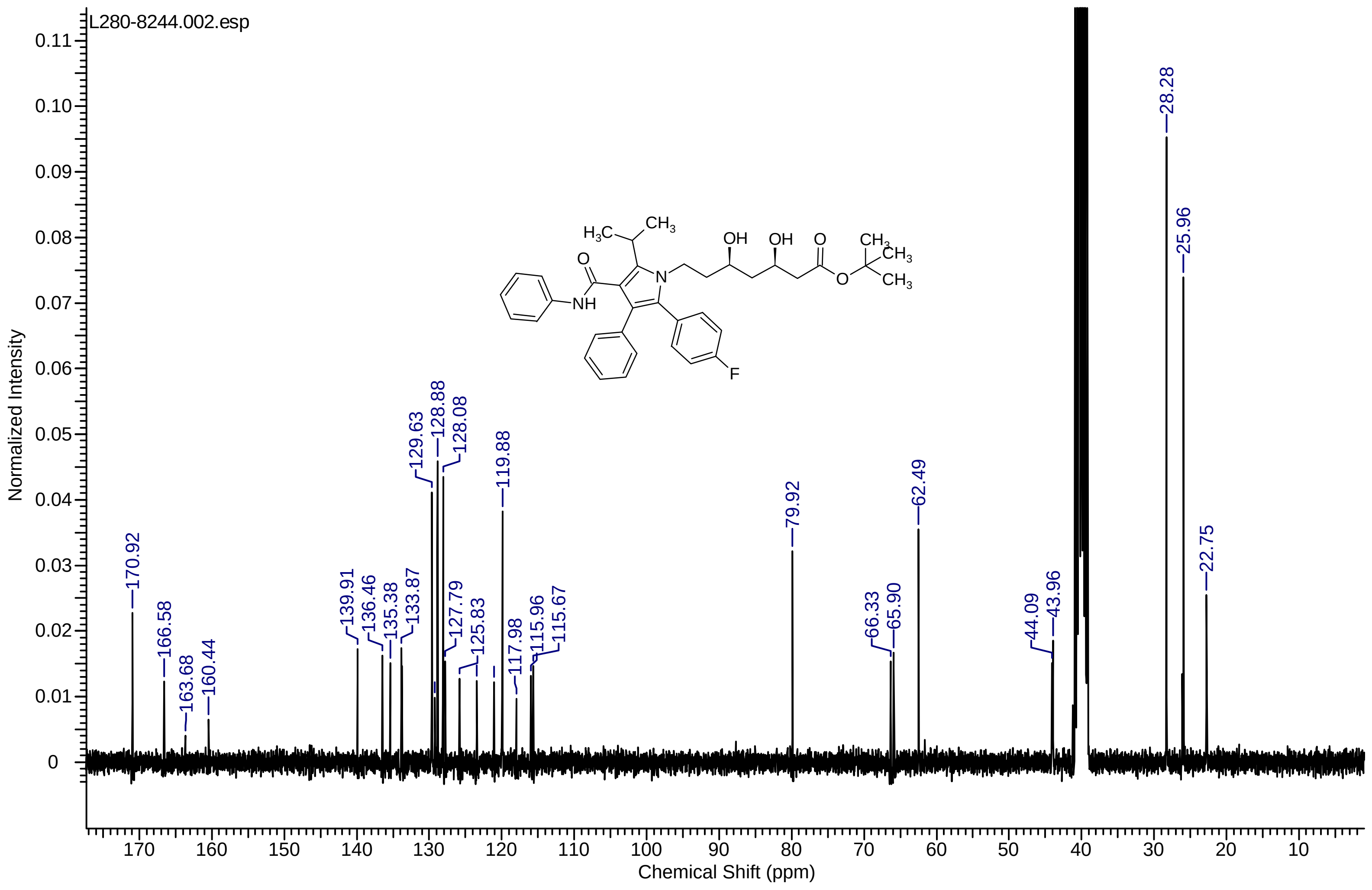

Compound **1**: ^{1}H NMR spectrum

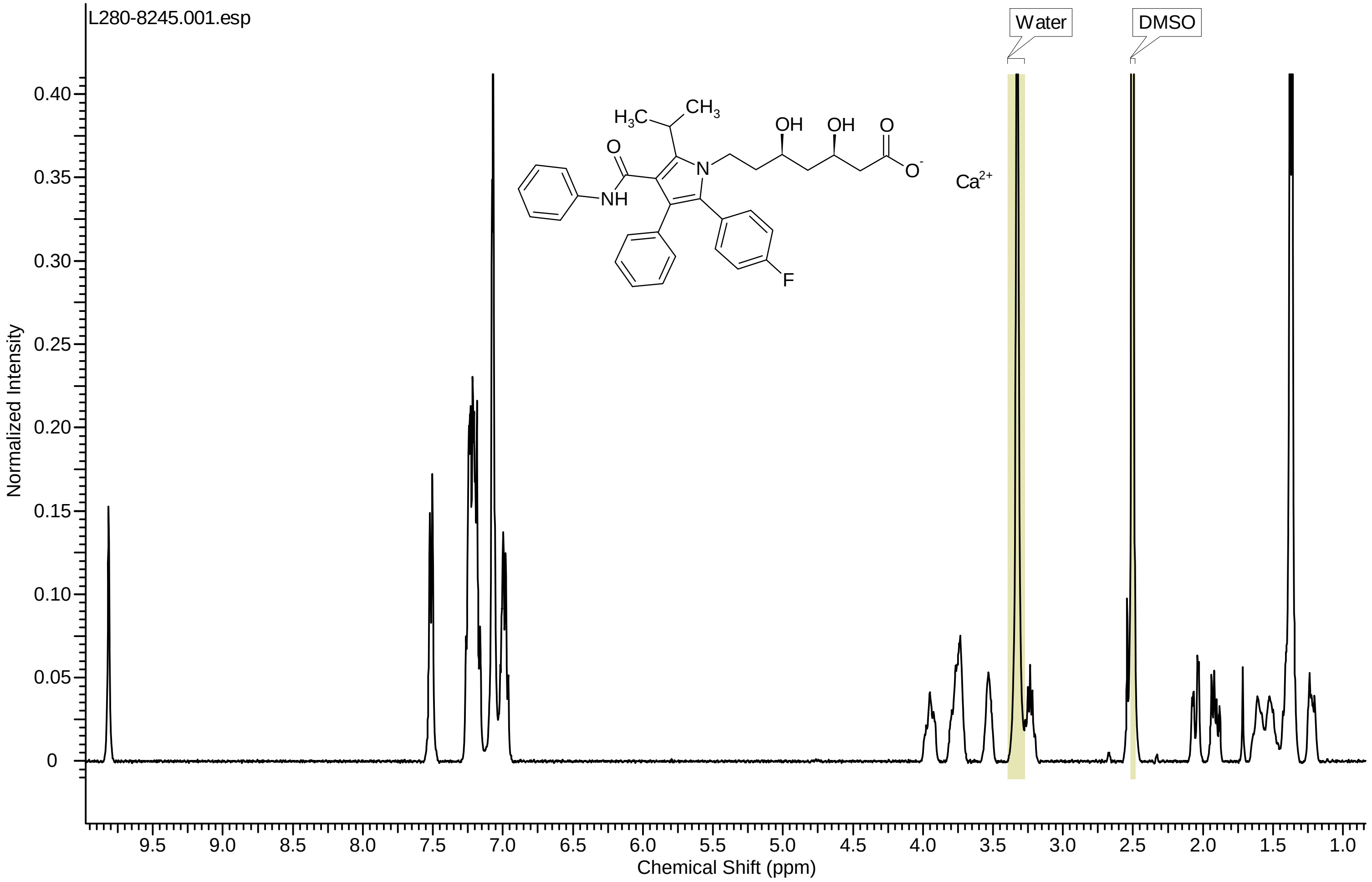

Compound **1**: ^{13}C NMR spectrum

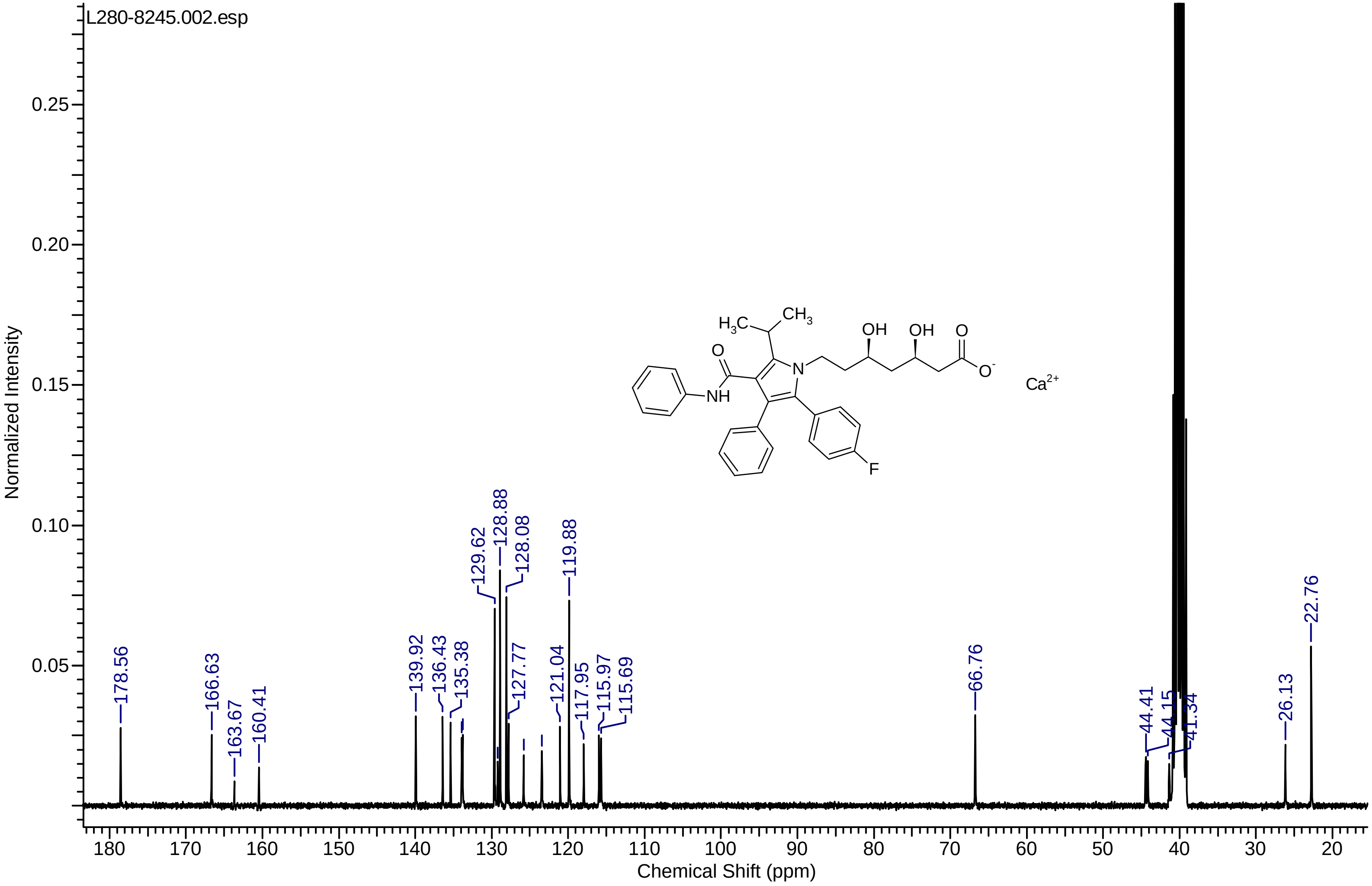

Quality control: impurity detection (HPLC)	
Chromatography conditions	
Column	Luna C-18(2) 100A (5 µm), 250×4,6 мм Phenomenex
Column temperature	25 ºC
Flow rate	1.0 mL/min
Run time	60 min
Injection volume	15 µL
Detector	UV, 250 nм
Mobile phase	Varying, according to the gradient elution program

SOLUTION PREPARATION

pH 5 buffer solution: ammonium acetate (3.19 g) was weighed out, dissolved in de-ionized water (950 mL). pH was adjusted to 5.0 (using a pH meter) with 20% acetic acid and the volume was adjusted to 1,000 mL.

Mobile phase component A: acetonitrile and pH 5.0 buffer solution (33:67).

Mobile phase component B: acetonitrile (MeCN).

Mobile phase component C: tetrahydrofuran.

Solvent system for sample preparation: MeCN : pH 5.0 buffer solution (60:40)

Gradient elution program

Step	Time interval	Component A*, %	Component B*, %	Component C*, %
1	0 – 5 min	90	10	0
2	5 – 25 min	90 → 10	10 → 90	0
3	25 – 30 min	10	90	0
4	30 – 32 min	10 → 90	90 → 10	0
5	32 – 40 min	90	10	0

* all solvents were filtered through a 0.45 µm membrane filter and degassed.

Solution for analysis (C =0,5 mg/mL): the analyzed substance (25.00 mg) was placed in a 50 mL volumetric flask, dissolved in the solvent system (20 mL) and the total volume was adjusted to 50 mL.

Standard solution for measurement: the solution for analysis (1.0 mL) was placed in a 100 mL volumetric flask and the total volume was adjusted to 100 mL.

Calculations:

Any impurity content in the substance (Xi) was calculated using the following formula:

$$X = \frac{S_i}{S_{av} \cdot F}$$

where S_i – Peak area in the analyzed sample solution;

S_{cp} – Atorvastatin peak area the comparator solution chromatogram;

F – Sensitivity coefficient for the impurity.

When calculating the impurity content the peaks present in the blank chromatogram are not taken into account.

Comparator solution chromatogram

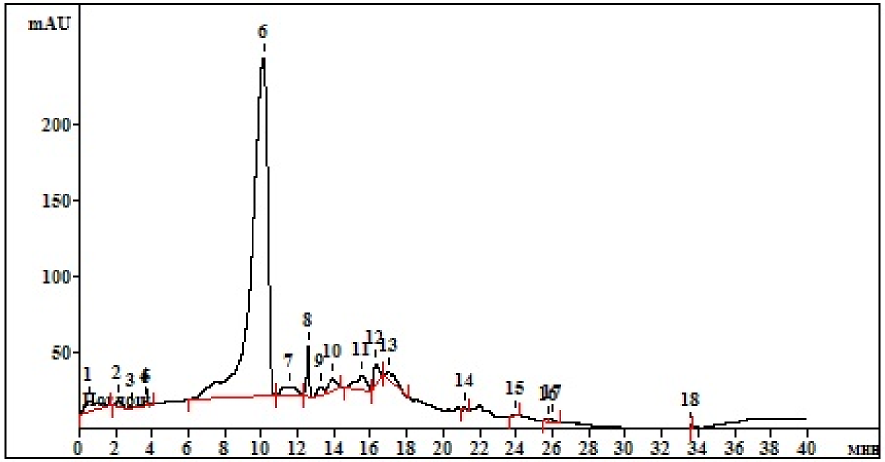

Пик	Время мин	Высота mAU	Площадь mAU*сек
1	0.5068	6.80	359.03
2	2.133	4.08	89.36
3	2.885	1.05	36.70
4	3.624	3.25	43.45
5	3.8	2.04	24.21
6	10.13	222.79	13436.09
7	11.5	5.74	324.72
8	12.58	32.97	326.48
9	13.28	4.72	110.89
10	13.89	7.77	201.79
11	15.53	9.75	424.84
12	16.28	12.72	241.85
13	16.98	4.45	160.85
14	21.13	1.67	15.90
15	23.96	0.81	11.76
16	25.76	1.45	17.84
17	25.94	2.25	42.87
18	33.61	2.29	8.41

Analyzed solution chromatogram

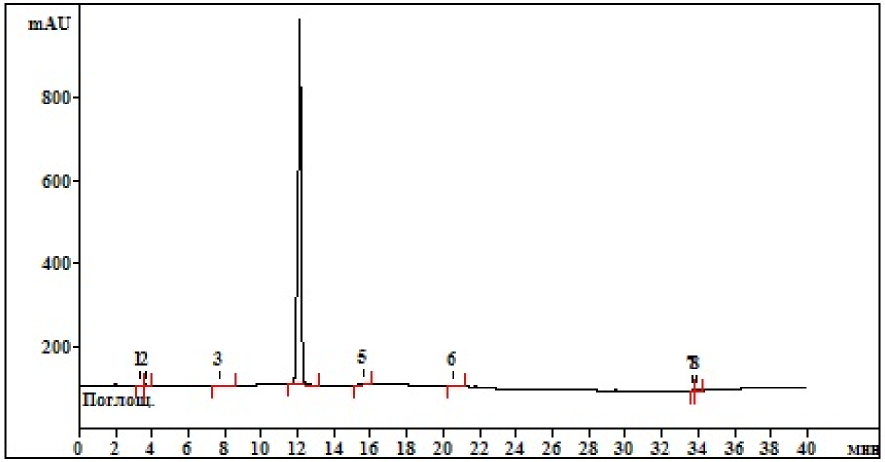

Пик	Время мин	Высота mAU	Площадь mAU*сек
1	3.288	1.35	14.98
2	3.638	1.76	17.98
3	7.701	1.52	61.24
4	12.12	879.22	10458.19
5	15.53	4.14	111.99
6	20.51	1.63	59.59
7	33.67	3.17	8.60
8	33.94	1.77	19.53

Results				
**Impurity**	**Pharmacopoeia requirement (max)**	**Relative retention time**	**Retention time, min**	**Impurity content, %**
Unidentified impurity 1	0.1 %	1.28	15.53	0.008
Unidentified impurity 2	0.1 %	1.69	20.51	0.004
Total impurities	1.0 %			0.012

Conclusion: atorvastatin calcium (batch number R34-0034) conforms to the purity requirements of European Pharmacopoiea 8.0.
